# bubbleHeatmap: an R package for visualization of Nightingale Health metabolomics datasets

**DOI:** 10.1093/bioadv/vbad123

**Published:** 2023-09-12

**Authors:** Ruth Boxall, Michael V Holmes, Robin G Walters

**Affiliations:** Clinical Trial Service Unit & Epidemiological Studies Unit, Nuffield Department of Population Health, University of Oxford, Oxford, United Kingdom; Medical Research Council Population Health Research Unit, Nuffield Department of Population Health, University of Oxford, Oxford, United Kingdom; Clinical Trial Service Unit & Epidemiological Studies Unit, Nuffield Department of Population Health, University of Oxford, Oxford, United Kingdom; Medical Research Council Population Health Research Unit, Nuffield Department of Population Health, University of Oxford, Oxford, United Kingdom; Clinical Trial Service Unit & Epidemiological Studies Unit, Nuffield Department of Population Health, University of Oxford, Oxford, United Kingdom; Medical Research Council Population Health Research Unit, Nuffield Department of Population Health, University of Oxford, Oxford, United Kingdom

## Abstract

**Summary:**

We present bubbleHeatmap, an R plotting package which combines elements of a bubble plot and heatmap to conveniently display two numerical variables for each data point across a categorical two dimensional grid. This has particular advantages for visualizing the 251 metabolomic measures produced by the automated, high-throughput, ^1^H-NMR-based platform provided by Nightingale Health, which includes 12 measures repeated across each of 14 lipoprotein subclasses. As these metabolomic profiles are currently available for large biobanks, we provide a figure template to aid the use of *bubbleHeatmap* in displaying results from analyses using these data.

**Availability and implementation:**

https://cran.r-project.org/web/packages/bubbleHeatmap

## 1 Introduction

Metabolomics is an emerging branch of systems biology which utilizes comprehensive molecular profiling of samples to gain greater insight into biological processes and pathways than is afforded by traditional biochemistry techniques. Developments in this field are likely to have considerable impact in the areas of biomarker discovery and precision medicine.

Nightingale Health (NH) provides a fully automated platform for deriving 251 quantitative metabolomics measures from a single blood sample using ^1^H-NMR technology (Soininen *et al.* 2015, Würtz *et al.* 2017), at a comparable cost to standard lipid clinical chemistry. These measures include concentration, composition, and relative proportions of lipoprotein subclasses, apolipoproteins, fatty acids, amino acids, and glycolysis-related metabolites. Over 1M samples have already been processed, with substantial capacity for processing of additional samples.

NH metabolomic profiles are already available for several large biobanks and global cohorts, including data on 275K UK Biobank (UKB) participants to date (with data on the remaining individuals to follow). The wide availability of datasets with NH measures in global cohorts facilitates collaborative research and is contributing to the identification of new biomarker associations across a range of diseases (Tynkkynen *et al.* 2018, Balling *et al.* 2020, Harris *et al.* 2020, Julkunen *et al.* 2023).

To date, NH profiles have been used in over 450 peer-reviewed publications. The presentation of findings from these studies varies considerably, which can hamper efforts to compare findings across these analyses. A set of 12 measures repeated across 14 lipoprotein subclasses make up around 75% of the available variables, and changes in associations of both across and within subclasses are of biological interest. However, the output from such analyses can pose a challenge to visualize effectively using traditional forest plots and the space required for forest plots hampers presentation of results for all metabolites in a single figure. Therefore, we have developed an R package *bubbleHeatmap* which displays results in a rectangular grid of colored “bubbles,” so that two numerical variables (e.g. effect size and *P*-value) can be represented by color and size, and grouped by categorical variables on the *x* and *y* axes. The package includes a template for presentation of both the latest 251 variable profile and legacy datasets containing 225 and 249 variables, and provides a simple workflow for generation of a single figure presenting results for the full set of NH variables. This should facilitate interpretation, dissemination, and comparison of research findings from different studies using NH metabolomics.

## 2 Plotting package and workflow

*bubbleHeatmap* is based on the R grid graphics system. Plots are returned as modifiable graphical objects (grobs) which can be output to a Cairo graphics device with gradient fill support using the grid.draw() function.

The first step in producing a figure presenting analyses using NH datasets is to align a results file with a template dataframe for either 251 or 225 variables. This can be done manually or using supplied merge keys (e.g. UKB Field IDs). The templates provide the information to arrange results into the 10 grids that make up the figure, including descriptive row and column names. The entire figure grob can then be produced in a single step using the *metabFigure()* function, or in four steps providing more modification options. All elements can be further edited prior to output, allowing unlimited customization.

The core *bubbleHeatmap()* plotting function has several other optional elements, such as labeled brackets to identify specific rows or columns of plots. The *bubbleHeatmap* framework can readily be customized to use alternative plot grids from user-defined data frames, e.g. to compare key variables from multiple analyses, or to display results for entirely different types of data unrelated to NH. Functions to combine and arrange plots allow creation of flexible multiplot layouts. Additional examples can be found in the function documentation and package vignette.

## 3 Package data

The sample data included in this package represent associations between NH metabolomic measures and a genetic score (GS) for cholesterol ester transfer protein (CETP) (Millwood *et al.* 2018). CETP transfers esterified cholesterol from high-density lipoprotein (HDL) to apolipoprotein B-containing lipoproteins in exchange for triglycerides. This process is a key component of the reverse cholesterol transport pathway, which modulates the return of cholesterol from peripheral cells such as macrophages to the liver, where it can be redistributed or excreted. Lower activity of CETP activity is associated with higher levels of HDL cholesterol (HDL-C) which has led to interest in CETP as a therapeutic target (Schmidt *et al.* 2021, Nicholls *et al.* 2022).

The provided data comprise the effect estimate and standard error, *P*-value, and -log_10_(*P*-value) for the association of the 225 metabolomics traits with a CETP GS, scaled to 10 mg/dl higher levels of HDL-C, in 4657 individuals from the China Kadoorie Biobank (CKB), a prospective study of Chinese adults from 10 distinct areas of China (Chen *et al.* 2011, Walters *et al.* 2023). The CETP analysis has been previously published (Millwood *et al.* 2018) but, due to the limitations of forest plots, only a subset of the metabolomics associations with the CETP GS were presented in the original paper.

## 4 Results

**Figure 1.**
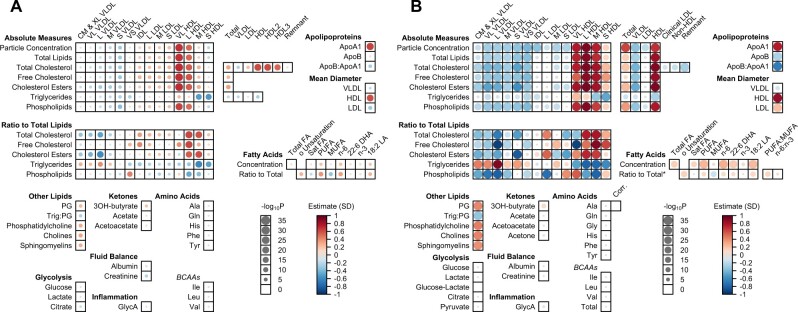
Comparison of CETP metabolomics association analyses. Association of CETP gene score with NMR metabolomics. (A) In China Kadoorie Biobank for 225 variables (Millwood *et al.* 2018). (B) In UK Biobank for 249 variables (Richardson *et al.* 2022). In each case, results are scaled to the effect corresponding to a 1 SD increase in HDL-C due to a genetically predicted decrease in CETP function. The *P*-value scale is capped at 10^−35^ for the UKB analysis. *Ratios are to total fatty acids except where specified.

Figure 1 shows two figure layouts generated using this package. Figure 1A uses the CETP GS association data provided with the package and demonstrates the 225 variable layout. Using the data as provided, this figure can be produced using a single line of code, as described in the package vignette. Figure 1B demonstrates the 251 variable layout, using results for 249 variables from a similar study conducted in UKB (Richardson *et al.* 2022); these data are not included with our package but are available for download as part of the original publication. This figure illustrates how *bubbleHeatmap* enables clear and accessible visualization of the associations of a variable of interest (i.e. CETP GS) with the full range of metabolites available from NH. Patterns in the main blocks are easily discernible across lipoprotein subclasses (rows) and within each subclass (columns), and remaining data points are meaningfully grouped according to function. Lipoprotein-related measures are shown at the top of the figure to the right of the main block, with fatty acids below. Blocks relating to Other Lipids, Glycolysis, Ketones, Fluid Balance, Inflammation, and Amino Acids are placed to the bottom left. The figure also demonstrates how this efficient display of results from NH datasets facilitates comparisons between analyses, in this case highlighting how the direction of association is inconsistent between the two studies for the ratio of free cholesterol to total lipids in very large HDL, and for mean low-density lipoprotein (LDL) diameter. In UKB, there is a strong inverse association with ratio of free cholesterol to total lipids in Large very low-density lipoprotein (VLDL) that is not seen in CKB. There are also differences in the associations of particle concentration, cholesterol, and phospholipids in medium HDL, and the ratio of phospholipids to total lipids in VLDL.

**Figure 2.**
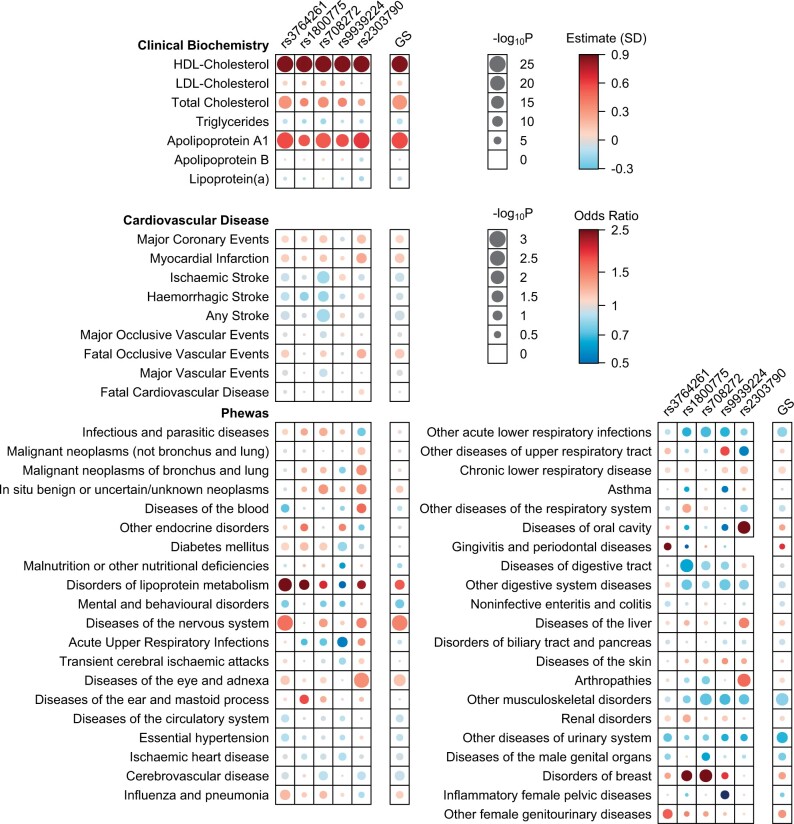
Association of CETP genetic variants with lipids, lipoproteins, and disease outcomes. Association in China Kadoorie Biobank of CETP gene score and its individual constituent SNPs with clinical lipid biochemistry measures and cardiovascular diseases, and from phenome-wide assocation analysis (Millwood *et al.* 2018). In each case, results are scaled to the effect corresponding to a 1 SD increase in HDL-C due to a genetically-predicted decrease in CETP function. The *P*-value scale is capped at 10^−25^ for the biochemistry variables. *Note*: SNP weights for the GS were from a multivariate model, and so are not directly comparable to results for univariate weights for individual SNPs.

Figure 2 illustrates how the package can be applied to other types of data to enable easy visualization of large sets of results, requiring only appropriately formatted results with a user-defined grid layout specification. It shows results from the same paper (Millwood *et al.* 2018) for the associations of the CKB CETP GS and its individual constituent SNPs with clinical biochemistry measures of lipids and lipoproteins and with cardiovascular diseases (CVD), together with association results from a phenome-wide scan (PheWAS) across a wide range of disease outcomes. Due to space considerations in the original paper, the biochemistry and CVD results were only presented for the GS and one functional SNP (rs2303790) in the main text, with results for other variants provided in a supplementary table. The PheWAS results did not reach the pre-specified threshold to denote statistical significance and were presented for the GS only, in a supplementary figure. Using the *bubbleHeatmap* enables all these results to be displayed in a single figure, and highlights where GS association results are inconsistent across different SNPs, for example how the apparent association of the GS with diseases of eye and adnexa is driven primarily by the functional SNP.
